# Antitumor Mechanisms of Curcumae Rhizoma Based on Network Pharmacology

**DOI:** 10.1155/2018/4509892

**Published:** 2018-02-05

**Authors:** Yan-Hua Bi, Li-hua Zhang, Shao-jun Chen, Qing-zhi Ling

**Affiliations:** ^1^The Children's Hospital, School of Medicine, Zhejiang University, Hangzhou, China; ^2^Department of Traditional Chinese Medicine, Zhejiang Pharmaceutical College, Ningbo, China; ^3^School of Medical Technology, Ningbo College of Health Science, Ningbo, China

## Abstract

Curcumae Rhizoma, a traditional Chinese medication, is commonly used in both traditional treatment and modern clinical care. Its anticancer effects have attracted a great deal of attention, but the mechanisms of action remain obscure. In this study, we screened for the active compounds of Curcumae Rhizoma using a drug-likeness approach. Candidate protein targets with functions related to cancer were predicted by reverse docking and then checked by manual search of the PubMed database. Potential target genes were uploaded to the GeneMANIA server and DAVID 6.8 database for analysis. Finally, compound-target, target-pathway, and compound-target-pathway networks were constructed using Cytoscape 3.3. The results revealed that the anticancer activity of Curcumae Rhizoma potentially involves 13 active compounds, 33 potential targets, and 31 signaling pathways, thus constituting a “multiple compounds, multiple targets, and multiple pathways” network corresponding to the concept of systematic actions in TCM. These findings provide an overview of the anticancer action of Curcumae Rhizoma from a network perspective, as well as setting an example for future studies of other materials used in TCM.

## 1. Introduction

Curcumae Rhizoma, known as* Ezhu *in Mandarin, is a traditional Chinese medication (TCM) commonly used in both traditional treatment and modern clinical care [1]. Its pharmacological actions, which include antitumor, antiplatelet aggregation, and antithrombosis, hepatoprotective, antioxidant, antimicrobial, and antiviral activities, have attracted a great deal of attention [1]. Among its activities, the antitumor effect has been most extensively studied. Together with the antithrombosis properties, the antitumor activity is relevant to the traditional concept of “activating qi and breaking blood stasis” [1]. TCM has developed over thousands of years and accumulated abundant clinical experience, resulting in the formation of a comprehensive and unique medical system [2]. Due to the complex nature of TCM, which is rooted in both medicinal herbs and an understanding of the human body, the mechanisms of action for many traditional medications remain unclear [3]. Consequently, it is difficult to dissect the antitumor mechanisms of Curcumae Rhizoma.

Network pharmacology, first proposed by Hopkins in 2007 [4], is an approach to drug design that encompasses systems biology, network analysis, connectivity, redundancy, and pleiotropy [5]. This paradigm is capable of describing complex interactions among biological systems, drugs, and diseases from a network perspective and in this sense shares the holistic perspective of TCM [2, 3, 6]. Network pharmacology has been increasingly applied to exploring the pharmacological mechanisms of TCM, including the effects of JiaWeixianJi Tang on inflammatory bowel disease [7], XiaoYao powder on anovulatory infertility [8], and so on. In our previous study of salvianolic acid A (SAA), an abundant water-soluble and potently antioxidative compound isolated from Danshen, a TCM, we constructed the drug-target-pathway network, providing a systematic and visual overview of possible molecular mechanisms and signaling pathways involving this compound [9]. Thus, network pharmacology represents a powerful tool for dissecting the mechanisms underlying the anticancer action of Curcumae Rhizoma.

In this study, we first computationally screened for active compounds of Curcumae Rhizoma by evaluating their drug-likeness (DL) and then predicted potential targets by an inverse docking method. Candidate targets were checked for cancer relevance by manual search of the PubMed database. Potential targets were submitted to analysis using the GeneMANIA and Database for Annotation, Visualization, and Integrated Discovery (DAVID) software. Finally, the pharmacological data were further integrated into compound-target and target-pathway networks. These networks provide a systematic overview of the antitumor effects of Curcumae Rhizoma.

## 2. Material and Methods

### 2.1. DL Evaluation via the TCMSP Server

The concept of DL, established from analyses of the physiochemical properties or/and structural features of existing small organic drugs and drug candidates, has been widely used to filter out compounds with undesirable properties, especially those with poor ADMET (absorption, distribution, metabolism, excretion, and toxicity) profiles [10]. A model for DL evaluation in the TCMSP database, based on molecular descriptors and the Tanimoto coefficient, was constructed by Ru's research group (http://ibts.hkbu.edu.hk/LSP/tcmsp.php) [11]:

(1) $$\begin{array}{c} T\left(\;A,B\;\right)=\frac{A\cdot B}{{\left\|A\right\|}^{2}+{\left\|B\right\|}^{2}-A\cdot B}, \end{array}$$

where *A* is a molecular descriptor of a given herbal ingredient and *B* represents the average of this property over all molecules in the DrugBank database [11, 12]. In this study, compounds with DL ≥ 0.18 were chosen for further investigation.

### 2.2. Computational Target Identification by PharmMapper and Data Mining

PharmMapper is an online tool for drug-target identification using a pharmacophore mapping approach [13]. It can identify potential protein targets for molecules of interest (drugs, natural products, or other newly discovered compounds) [13]. In this study, sdf files of all interesting compounds from Curcumae Rhizoma were downloaded from the TCMSP database and uploaded individually to the PharmMapper server. During this procedure, “Human protein targets only (v2010, 2241)” was selected, and other parameters were set to default values.

From the PharmMapper results, the top five targets of every interesting compound were chosen, and duplicates were merged. Next, each target name was used to search PubMed along with the keyword “cancer/tumor,” allowing elimination of protein targets that were irrelevant to cancer. The remaining proteins were considered as candidate targets in subsequent analyses.

### 2.3. Analysis by GeneMANIA

The GeneMANIA server can generate hypotheses about gene function, analyze gene lists, and prioritize genes for functional assays [14]. After selection of* Homo sapiens *as the organism, interesting genes from the screen were entered into the GeneMANIA search bar, and the output was downloaded.

### 2.4. GO and Pathway Analysis and Network Construction

DAVID, a powerful tool for network biology, comprises an integrated biological knowledge base and analytic tools aimed at systematically extracting biological meaning from large lists of genes or proteins [15]. Potential targets were uploaded to the DAVID 6.8 server (https://david.ncifcrf.gov/home.jsp), and GO and KEGG pathway information was acquired. To achieve a systematic understanding of the complex relationships among compounds, targets, and diseases, compound-target-pathway networks were constructed and analyzed in Cytoscape 3.3.

## 3. Results

### 3.1. Screening for Active Compounds

A total of 81 compounds (as displayed in Table S1) from Curcumae Rhizoma were selected from the TCMSP database, and active components were evaluated using a DL approach. This analysis yielded eight compounds, representing about 10% of the original number. Four additional compounds, curcumol, beta-elemene, curcumadiol, and germacrone, had low DL scores but were previously established as important components of Curcumae Rhizoma and have been confirmed to exert potent antitumor activity. We also selected curcumin, which has a higher DL score but has been historically neglected, for further consideration. Thus, 13 active compounds were ultimately chosen for further investigation.

### 3.2. Drug-Target Prediction and Check

**Figure 1:**
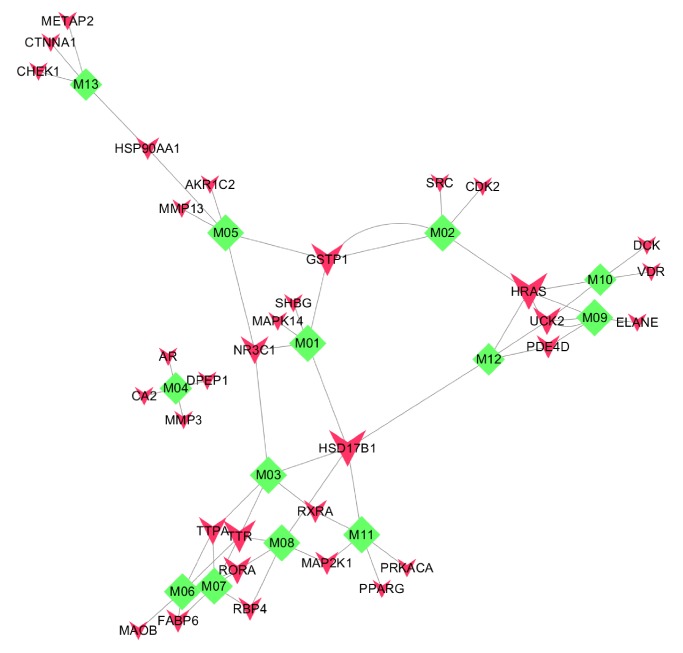
Compound-target network. Green diamond: compound; red inverted triangle: protein target; edge: interaction between a compound and a protein.

From the PharmMapper results, we obtained the top five potential targets for all 13 compounds, yielding 34 targets after deletion of duplicates. We then searched these targets in the PubMed database and eliminated one of the candidate targets on the grounds that it was not relevant to cancer. In the end, 33 targets were identified for 13 compounds of Curcumae Rhizoma, and their official names were obtained from the UniprotKB database. These compounds were subjected to further characterization. The compound-target network is shown in Figure 1.

### 3.3. GeneMANIA Analysis

**Figure 2:**
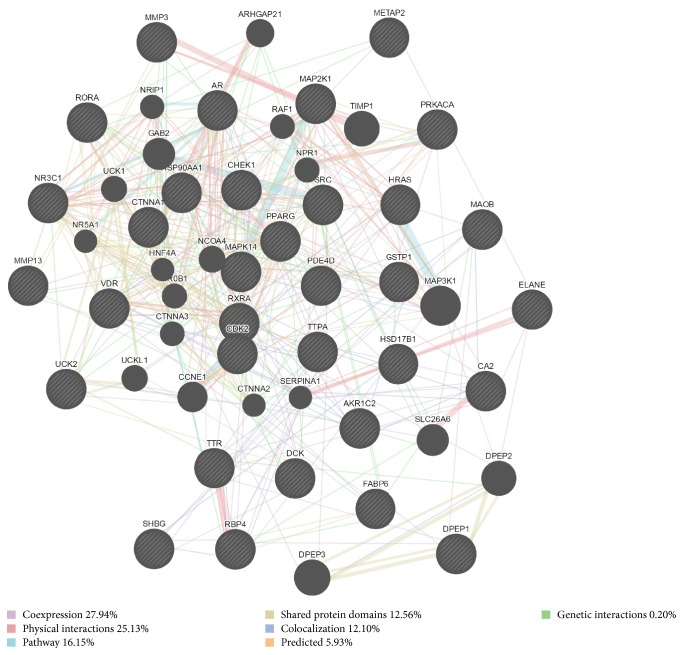
Network of potential targets analyzed using GeneMANIA. Black nodes: target proteins; colored lines: different interactions.

GeneMANIA revealed that, among the 33 targets and their interacting proteins, 27.94% are coexpressed and 25.13% engage in physical interactions. Other results, including pathway information, shared protein domains, colocalization, and predicted and genetic interactions, are shown in Figure 2.

### 3.4. GO and KEGG Analysis and Network Construction

**Figure 3:**
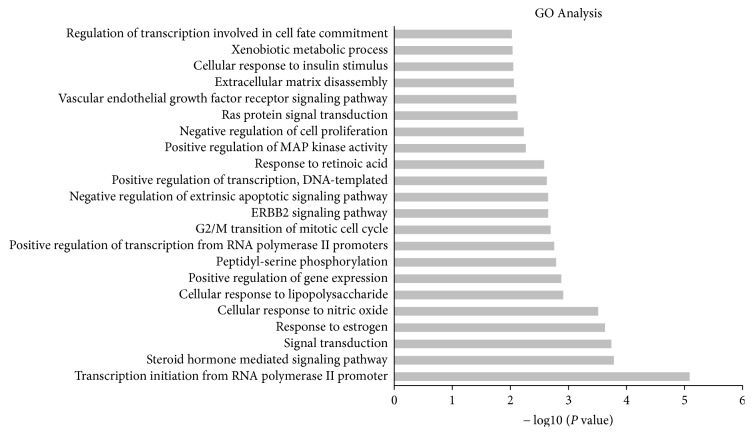
Gene Ontology (GO) analysis of targets. The *y*-axis shows significantly enriched Biological Process categories of the targets, and the *x*-axis shows the enrichment scores of these terms (*P* value < 0.05).

To further investigate the interaction network, we performed analyses using DAVID 6.8. As shown in Figure 3 and Table S2, the top five functions were transcription initiation from RNA polymerase II promoters, steroid hormone-mediated signaling pathway, signal transduction, response to estrogen, and cellular response to nitric oxide.

**Figure 4:**
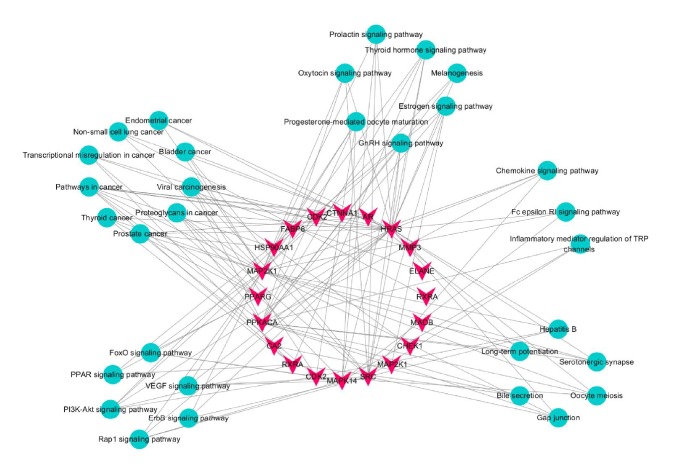
Target-pathway network. Red inverted triangle: protein target; cyan circle: pathway; edge: interaction between a target and a pathway.

**Figure 5:**
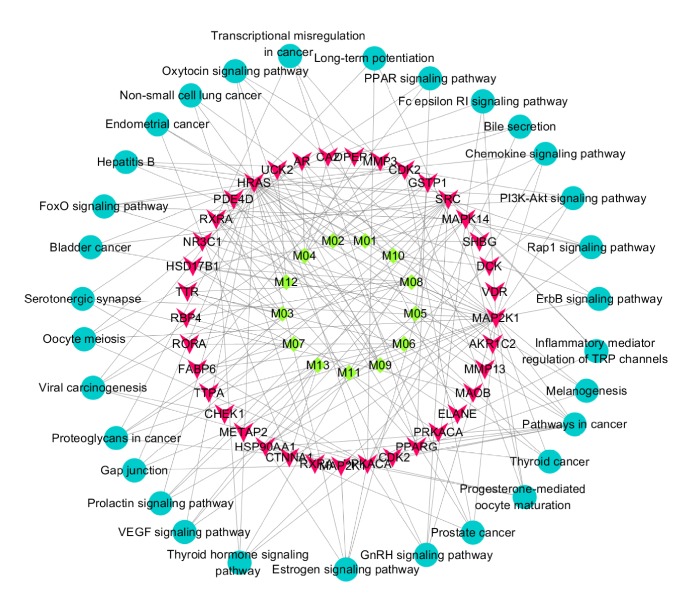
Compound-target-pathway network of Curcumae Rhizoma. Green diamond: compound; red inverted triangle: protein target; cyan circle: pathway.

As shown in Table 3, the 33 targets were involved in 31 KEGG pathways (*P* ≤ 0.05). The top five pathways were related to cancer, thyroid cancer, progesterone-mediated oocyte maturation, prostate cancer, and GnRH signaling. The target-pathway network is shown in Figure 4. Based on target identification and pathway analysis, we constructed a compound-target-pathway interaction network (Figure 5) with 81 nodes and 188 edges, with nodes corresponding to compounds, targets, or pathways and edges indicating interactions.

## 4. Discussion

DL relates simple molecular properties, such as molecular weight (MW), physicochemical properties, and the number of rotatable bonds or aromatic rings, to the potential success of a drug discovery objective, generally in regard to suitable pharmacokinetics and safety [16]. High DL reflects a greater likelihood of a compound becoming a drug. Because drug discovery from a given candidate compound is not guaranteed to succeed, the DL approach has been widely used to filter out compounds with undesirable properties, especially those with poor ADMET profiles [10, 16]. For DrugBank compounds, average DL ≥ 0.18 has been used as a criterion for screening of bioactive compounds in systematic pharmacology-based analyses of TCM [11, 12]. As shown in Table 1, nine of the compounds we investigated fit this requirement; in addition, four compounds with low DL values were also selected for study. Several of these compounds were previously shown to exert antitumor effects. For example, curcumin (M02 in Table 1) is widely used against tumors and suppresses the growth of gastric tumor cells [17] and human glioma cells [18]. Similarly, curcumol (M01) induces apoptosis in SPC-A-1 human lung adenocarcinoma cells [19], hederagenin (M06) exerts cytotoxicity in human breast and lung cancer cells [20], and demethoxycurcumin (M10) inhibits the growth of human epithelia ovarian cancer cells [21]. Thus, many compounds from Curcumae Rhizoma may have antitumor effects, consistent with the synergistic effects of multiple components of TCMs.

Target “fishing” or target identification is an important start step in modern drug development. This process entails investigating the mechanism of action of bioactive small molecules by identifying their interacting proteins [22]. PharmMapper has been widely used for computational target identification, which can provide the top 300 candidate targets for the query compound in default [13]. For data visualization, we reduced the amount of data and kept the top 5 candidates for every molecule. Several of the potential targets of active compounds from Curcumae Rhizoma have been identified in other studies. For example, curcumin interrupts the interaction between the androgen receptor (AR) and Wnt/*β*-catenin signaling pathway in LNCaP prostate cancer cells [23]. On the other hand, germacrone exerts antiandrogenic effects in* in vitro* and* in vivo* assays but does not bind AR [24]. CDK2 was identified as a direct target of curcumin in colon cancer cells [25], and germacrone induces G1 phase arrest, associated with a significant decrease in expression of cyclin D1 and CDK2 and elevated expression of p21 [26]. Curcumol inhibits the proliferation of HepG2 cells* in vitro* and induces G1 arrest by activating the p53 and pRB pathways, whose downstream targets include the genes encoding cyclin A1, CDK2, CDK8, p21WAF1, and p27KIP1 [27]. Moreover, curcumin inhibits human cytomegalovirus by downregulating heat shock protein 90 (Hsp90) [28], whereas beta-elemene inhibits formation of the Hsp90/Raf-1 complex, thereby inducing apoptosis in glioblastoma cells [29]. Thus, the antitumor effects of Curcumae Rhizoma are mediated by multiple targets, often interacting synergistically (Table 2).

GO and pathway analyses were conducted using Cytoscape 3.3. As shown in Figure 4, the network also indicated that Curcumae Rhizoma has multiple targets and implies that it therefore exerts multiple antitumor pharmacological effects. The 31 significant pathways (*P* ≤ 0.05) identified in this study included several related to cancer; indeed, the top two were “pathways in cancer” and “thyroid cancer” (as listed in Table 3). As shown in Figure 4, Curcumae Rhizoma is predicted to have effects on multiple cancers, including endometrial cancer, non-small-cell lung cancer, bladder cancer, thyroid cancer, and prostate cancer. Consistent with this, previous studies show that Curcumae Rhizoma or its active compounds are effective against cancer. Consistent with this, and as described above, several previous studies show that Curcumae Rhizoma or its active compounds are effective against cancer [19, 21, 23, 25, 30].

Steroid hormones play important roles in cancer-associated proliferation, apoptosis, migration, and invasion, and so on [30]. Some cancers, for example, breast tumors, are hormone-responsive cancers in which steroid hormones exert their mitogenic effects by binding to estrogen, progesterone, and ARs, highlighting pathways that may be instrumental in the etiology of breast cancer [31]. As shown in Figure 4, Curcumae Rhizoma can act through hormone-related pathways, including those mediated by estrogen, prolactin, and oxytocin. Furanodiene, a natural product isolated from Curcumae Rhizoma, stimulates the anticancer effects of doxorubicin in ER*α*-negative breast cancer cells [32]. Other signaling pathways (Figure 4), such as those involved in inflammation and immunity, also play important roles in the anticancer effect.

## 5. Conclusions

Eight of the thirteen active compounds were selected by a DL strategy, and their potential targets were identified by PharmMapper and analyzed by network-related tools. The results revealed that the 13 active compounds exert their antitumor effects via 33 targets in 31 pathways. This is consistent with the TCM concept of “multiple compounds, multiple targets, and multiple effects.” Although further experiments are needed to provide support for our findings, this study provides a systematic view of the potential anticancer mechanisms of Curcumae Rhizoma from a network-based perspective.

## Figures and Tables

**Table 1:** 13 candidate compounds and their DL values.

ID	Name	DL
M01	Curcumol	0.13
M02	Curcumin	0.41
M03	Beta-elemene	0.06
M04	Curcumadiol	0.1
M05	Germacrone	0.07
M06	Hederagenin	0.75
M07	Alexandrin	0.63
M08	Difurocumenone	0.61
M09	TNP00001	0.41
M10	Demethoxycurcumin	0.33
M11	Wenjine	0.27
M12	Bisdemethoxycurcumin	0.26
M13	(1S,10S),(4S,5S)-Germacrone-1(10),4-diepoxide	0.18

**Table 2:** Information about cancer-related targets of Curcumae Rhizoma.

Name	Gene	UniprotKB
Aldo-keto reductase family 1 member C2	*AKR1C2*	P52895
Alpha-tocopherol transfer protein	*TTPA*	P49638
Amine oxidase [flavin-containing] B	*MAOB*	P27338
Androgen receptor	*AR*	P10275
cAMP-dependent protein kinase catalytic subunit alpha	*PRKACA*	P17612
cAMP-specific 3,5-cyclic phosphodiesterase 4D	*PDE4D*	Q08499
Carbonic anhydrase 2	*CA2*	P00918
Catenin alpha-1	*CTNNA1*	P35221
Cell division protein kinase 2	*CDK2*	P24941
Collagenase 3	*MMP13*	P45452
Deoxycytidine kinase	*DCK*	P27707
Dipeptidase 1	*DPEP1*	P16444
Dual specificity mitogen-activated protein kinase kinase 1	*MAP2K1*	Q02750
Estradiol 17-beta-dehydrogenase 1	*HSD17B1*	P14061
Gastrotropin	*FABP6*	P51161
Glucocorticoid receptor	*NR3C1*	P04150
Glutathione S-transferase P	*GSTP1*	P09211
GTPase H-Ras	*HRAS*	P01112
Heat shock protein HSP 90-alpha	*HSP90AA1*	P07900
Leukocyte elastase	*ELANE*	P08246
Methionine aminopeptidase 2	*METAP2*	P50579
Mitogen-activated protein kinase 14	*MAPK14*	Q16539
Nuclear receptor ROR-alpha	*RORA*	P35398
Peroxisome proliferator-activated receptor gamma	*PPARG*	P37231
Proto-oncogene tyrosine-protein kinase Src	*SRC*	P12931
Retinoic acid receptor RXR-alpha	*RXRA*	P19793
Retinol-binding protein 4	*RBP4*	P02753
Serine/threonine-protein kinase Chk1	*CHEK1*	O14757
Sex hormone-binding globulin	*SHBG*	P04278
Stromelysin-1	*MMP3*	P08254
Transthyretin	*TTR*	P02766
Uridine-cytidine kinase 2	*UCK2*	Q9BZX2
Vitamin D3 receptor	*VDR*	P11473

**Table 3:** KEGG pathway analysis of potential targets, using DAVID6.8 (*P* < 0.05).

Term	*P* value
Pathways in cancer	4.70*E* − 05
Thyroid cancer	1.40*E* − 04
Progesterone-mediated oocyte maturation	2.40*E* − 04
Prostate cancer	2.50*E* − 04
GnRH signaling pathway	2.90*E* − 04
Estrogen signaling pathway	4.00*E* − 04
Thyroid hormone signaling pathway	6.80*E* − 04
VEGF signaling pathway	1.30*E* − 03
Prolactin signaling pathway	2.00*E* − 03
Gap junction	3.70*E* − 03
Proteoglycans in cancer	5.30*E* − 03
Viral carcinogenesis	5.80*E* − 03
Oocyte meiosis	6.80*E* − 03
Serotonergic synapse	7.20*E* − 03
Bladder cancer	9.50*E* − 03
FoxO signaling pathway	1.20*E* − 02
Hepatitis B	1.50*E* − 02
Endometrial cancer	1.50*E* − 02
Non-small-cell lung cancer	1.70*E* − 02
Oxytocin signaling pathway	1.90*E* − 02
Transcriptional misregulation in cancer	2.20*E* − 02
Long-term potentiation	2.30*E* − 02
PPAR signaling pathway	2.40*E* − 02
Fc epsilon RI signaling pathway	2.50*E* − 02
Bile secretion	2.50*E* − 02
Chemokine signaling pathway	2.90*E* − 02
PI3K-Akt signaling pathway	3.40*E* − 02
Rap1 signaling pathway	3.90*E* − 02
ErbB signaling pathway	3.90*E* − 02
Inflammatory mediator regulation of TRP channels	4.80*E* − 02
Melanogenesis	5.00*E* − 02

## Supplementary Materials

Supplementary 1 Table S1: 81 compounds of Curcumae Rhizoma.

Supplementary 2 Table S2: total predicted Gene Ontology (GO) Biological Process (BP) for Curcumae Rhizoma using DAVID6.8.
